# Association of AGTR1 A1166C and CYP2C9∗3 Gene Polymorphisms with the Antihypertensive Effect of Valsartan

**DOI:** 10.1155/2022/7677252

**Published:** 2022-03-19

**Authors:** Yi Liu, Xiaomu Kong, Yongwei Jiang, Meimei Zhao, Peng Gao, Xiao Cong, Yongtong Cao, Liang Ma

**Affiliations:** Department of Clinical Laboratory, China-Japan Friendship Hospital, Beijing, China

## Abstract

**Background:**

The differences in the antihypertensive treatment with angiotensin type II receptor blockers (ARBs) may be attributed to polymorphisms in genes involving drug-targeted receptor and drug metabolism. The present study aimed to investigate whether the antihypertensive effect of the ARB drug valsartan was associated with angiotensin II type 1 receptor (*AGTR1*) gene polymorphism (A1166 C) and cytochrome P450 enzyme 2C9 (*CYP2C9*) gene polymorphism (*CYP2C9*∗3).

**Methods:**

281 patients with hypertension who received valsartan monotherapy in the past month were included in this retrospective study. Polymerase chain reaction-melting curve analysis was performed to genotype the *AGTR1* and *CYP2C9* gene polymorphisms. Based on the systolic blood pressure (SBP) and diastolic blood pressure (DBP) at the time of visit, the patients were divided into well-controlled group (*n* = 144, SBP/DBP <140/90 mmHg) and poorly controlled group (*n* = 137, SBP/DBP ≥140/90 mmHg).

**Results:**

Older age, decreased history of drinking, a higher proportion of mild-to-moderate hypertension, lower alanine aminotransferase levels, and higher high-density lipoprotein cholesterol levels were observed in the well-controlled group than the poorly controlled group. Higher frequencies of the C allele and AC + CC genotype of *AGTR1* A1166C were detected in the well-controlled than the poorly controlled patients (*P* = 0.005 and *P* = 0.006). After adjustment for demographic and environmental factors, the CC + AC genotype of *AGTR1* A1166C was markedly linked to better hypertension control with valsartan treatment compared to the AA genotype (odds ratio: 2.836, 95% confidence interval: 1.199–6.705, *P* = 0.018). No significant difference was observed in the allele or genotype distribution of *CYP2C9*∗3 polymorphism between well-controlled and poorly controlled patients.

**Conclusions:**

The current data suggested that the *AGTR1* A1166 C polymorphism may be associated with the antihypertensive effect of valsartan, and carriers with AC and CC genotypes may have a better antihypertensive efficacy response to valsartan treatment.

## 1. Introduction

Hypertension is a major risk factor for cardiovascular disease and end-stage renal damage, affecting about 40% of the adult population worldwide, leading to increased mortality [1–3]. A survey conducted between 2012 and 2015 revealed that 23.2% of Chinese adults and approximately 40% of adults aged 45 years and older had hypertension [4, 5]. Evidence from clinical studies have proven that efficient control of blood pressure (BP) reduces cardiovascular- and stroke-associated morbidity and mortality [6, 7]. However, hypertension is controlled in <10% of Chinese patients who receive antihypertension medication [8, 9].

Angiotensin type II receptor blockers (ARBs) are widely used for hypertension treatment globally, with well-established safety profiles, and are better tolerated than other classes of antihypertensive agents [10, 11]. Especially, valsartan has been leading China's ARB market from 2011 to 2017, with high treatment adherence in patients with hypertension based on the prescription information in 2014 [12, 13]. However, antihypertensive responses to ARBs differ from one patient to another and single nucleotide polymorphisms (SNPs) in genes involving drug-targeted receptors and drug metabolism are associated with this individual variation [14].

ARBs exhibit a BP-lowering effect by blocking angiotensin II receptor type1 (AGTR1), encoded by the *AGTR1* gene, and mediate cardiovascular effects of angiotensin II, such as vasoconstriction, increased BP, and myocardial contractility [15]. In addition, ARBs are mainly metabolized by the hepatic cytochrome P450 2C9 (CYP2C9) encoded by the *CYP2C9* gene and transformed into inactive/active forms [16, 17]. Several studies have shown that SNPs in *AGTR1* and *CYP2C9* genes contribute to the antihypertensive effect of ARBs [18–22]. Among these, two polymorphisms rs5186 of the *AGTR1* gene or *AGTR1* A1166C and rs1057910 of the *CYP2C9* gene or *CYP2C9*∗3 were extensively studied in patients using different ARB agents or from different genetic backgrounds; however, the results were conflicting [16, 17, 19, 22, 23]. In addition, the data on the effect of *CYP2C9* and *AGTR1* polymorphisms on the antihypertensive efficacy response to valsartan were limited.

The present study aimed to investigate whether the antihypertensive effect of angiotensin receptor antagonist valsartan is associated with polymorphisms *AGTR1* A1166C and *CYP2C9*∗3 in a Chinese population and provide information about the heterogeneity of antihypertensive drug responses, which would help the clinicians to make an informed decision about individualized medication for hypertension.

## 2. Materials and Methods

### 2.1. Study Design

This was a retrospective study of 281 Chinese patients, selected from 1682 patients with hypertension who visited China-Japan Friendship Hospital, Beijing, China, between January 2016 and December 2018. The present study was approved by the hospital Institutional Ethics Committee. Written informed consent was obtained from all individuals.

#### 2.1.1. Patients

**Figure 1:**
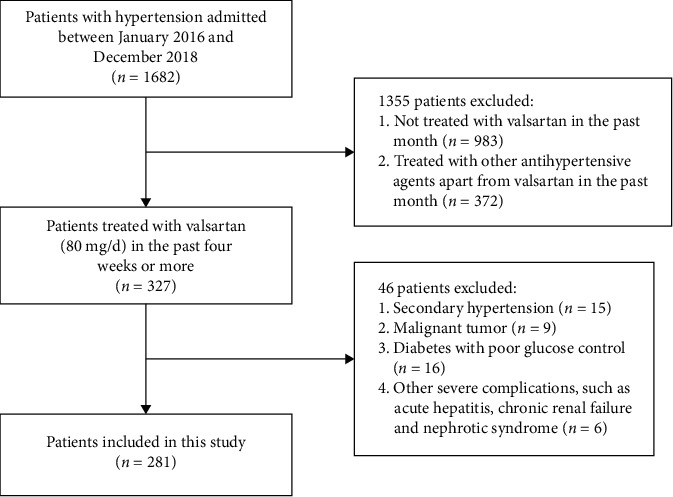
Study population flowchart.

The inclusion criteria were as follows: (1) a definite diagnosis of hypertension (systolic BP, SBP ≥140 mmHg and diastolic BP, DBP ≥90 mmHg); (2) age >18 years and <65 years; and (3) antihypertensive treatment with valsartan 80 mg/day for at least 4 weeks before the visit to the hospital. The exclusion criteria were as follows: (1) secondary hypertension of any etiology; (2) poorly controlled diabetes, a history of malignant tumor, or severe complications, such as renal failure and nephritic syndrome; and (3) other drugs apart from valsartan used for antihypertensive treatment. The patient selection flowchart is presented in Figure 1.

Hypertension is classified as Grade 1, Grade 2, and Grade 3, and a BP control target of <140/90 mmHg (<130/80 mmHg for patients with diabetes or coronary heart disease) is recommended according to the 2018 guidelines of the European Society of Hypertension (ESH) and Chinese hypertension guidelines [24, 25]. In the present study, patients were divided into the well-controlled group and poorly controlled group, depending on whether they achieved the target BP levels.

#### 2.1.2. Genotyping SNPs

Genomic DNA was extracted from peripheral blood samples using the QIAamp DNA Blood Mini Kit (Qiagen, Hilden, Germany). The purity and quantity of the extracted DNA were measured on a NanoDrop 1000 spectrophotometer (ThermoScientific, Waltham, MA, USA). Polymerase chain reaction (PCR)-melting curve analysis was performed to genotype these SNPs using a commercial kit (RIQIgen Biotech Co., Ltd., Wuxi, China), including specific primers and fluorescent probes for *AGTR1* A1166C and *CYP2C9*∗3. The following thermocycling conditions were used for the PCR: pre-denaturation at 95°C for 2 min; 50 cycles of denaturing at 94°C for 30 sec, annealing at 56°C for 30 sec, and extension at 70°C for 30 sec. The subsequent melting analysis process included three steps: denaturation at 95°C for 30 sec; renaturation at 46°C for 60 sec followed by continuous fluorescence reading mode at 46–75°C with increasing temperature gradient at a rate of 0.3°C/s. The melting curves were analyzed on a Tianlong Gentier 96E PCR analysis system (Tianlong Science and Technology Co., Ltd., Xi'an, China) and matching software.

### 2.2. Statistical Analysis

Continuous variables were expressed as median (interquartile range), and categorical variables were presented as frequencies and percentages. The between-group data were compared using Mann–Whitney *U* test for continuous variables and *χ*^2^ test for categorical variables. A goodness of fit *χ*^2^ test was used to evaluate whether the genotype distributions of *AGTR1* A1166C and *CYP2C9*∗3 polymorphisms were in accordance with Hardy–Weinberg equilibrium. The differences in genotype frequencies between groups were compared by *χ*^2^ test or Fisher's exact test. Because the CC genotype of *AGTR1* A1166C polymorphism was extremely rare, a dominant genetic model (AA genotype *vs*. AC + CC genotype) was used in the logistic regression analysis. Univariable logistic regression analysis was used to evaluate the association between gene polymorphisms and the response to valsartan treatment, and the data were presented as unadjusted odds ratios (ORs) with 95% confidence intervals (CIs). Multivariate logistic regression was used to determine the factors associated with the antihypertensive efficacy of valsartan with adjustment for covariates: age, gender, body mass index (BMI), grade of hypertension, and history of drinking and smoking. The data were presented as adjusted ORs with 95% CIs. Statistical analysis was performed using SPSS version 20.0 (SPSS Inc., Chicago, USA), and *P* < 0.05 was considered statistically significant.

## 3. Results

### 3.1. General Characteristics

There were 144 patients in the well-controlled group (achieving target BP levels) and 137 patients in the poorly controlled group (failing to achieve target BP levels). The demographic and clinical characteristics of the two groups are summarized in Table 1. Advanced age, less history of drinking, lower alanine aminotransferase (ALT) levels, and higher high-density lipoprotein cholesterol (HDL-C) levels were observed in the well-controlled group than the poorly controlled group (Table 1). Moreover, the grade of hypertension differed significantly between the two groups (*P* = 0.001). No differences were detected in other variables, such as gender, BMI, history of smoking, levels of total cholesterol, triglyceride, low-density lipoprotein cholesterol (LDL-C), creatinine, and estimated glomerular filtration rate (eGFR) between the two groups (Table 1).

### 3.2. Genotype Distributions of *AGTR1* A1166C and *CYP2C9*∗3

The distribution of allele frequencies of *AGTR1* A1166C and *CYP2C9*∗3 was in accordance with the Hardy–Weinberg equilibrium in both well-controlled and poorly controlled groups. The genotype and allele distributions of gene polymorphisms between the two groups are shown in Table 2. The genotype and allele frequencies of *AGTR1* A1166C polymorphism differed between the well-controlled and poorly controlled group (*P* = 0.006 and *P* = 0.005, respectively), while no significant difference was observed in the genotype or allele frequency of *CYP2C9*∗3 between the two groups (*P* = 0.239 and *P* = 0.250, respectively). Only one patient carried the CC genotype of *AGTR1* A1166C polymorphism; therefore, a dominant genetic model for *AGTR1* A1166C (AA genotype *vs*. AC + CC genotype) was used in the subsequent analysis.

### 3.3. Association of Gene Polymorphisms with the Antihypertensive Effect of Valsartan

The association of *AGTR1* A1166C and *CYP2C9*∗3 genotypes with the response to valsartan treatment was evaluated by univariate and multivariate regression analyses (Table 3). In terms of *AGTR1* polymorphism, AC + CC genotype significantly increased the control rate of hypertension in patients with valsartan treatment compared to the AA genotype (AC + CC *vs*. AA: OR 2.988, 95% CI 1.340–6.661, *P* = 0.007). However, in terms of *CYP2C9* polymorphism, ∗1/∗3 genotype did not affect the hypertension control rate in valsartan treatment (∗1/∗3 *vs*. ∗1/∗1: OR 0.607, 95% CI 0.263–1.402, *P* = 0.242). The results were similar after adjusting for age, gender, BMI, grade of hypertension, and history of drinking and smoking. In addition, age was also an independent factor affecting the response to valsartan treatment in the multivariate analysis (OR 1.035, 95% CI 1.004–1.067, *P* = 0.028) (Table 3).

Considering the potential synergistic effect of *AGTR1* A1166C and *CYP2C9*∗3 polymorphisms, the combined effect of *AGTR1* A1166C and *CYP2C9*∗3 genotypes on the antihypertensive efficacy response to valsartan was further analyzed (Table 4). When the genotype of *AGTR1* TT and *CYP2C9* ∗1/∗1 was used as the reference, the combined genotype of *AGTR1* AC + CC and *CYP2C9* ∗1/∗1 showed a higher rate of hypertension control with valsartan treatment in the unadjusted and adjusted statistical models (unadjusted: OR 2.953, 95% CI 1.267–6.880, *P* = 0.012; adjusted: OR 3.028, 95% CI 1.225–7.486, *P* = 0.016). Also, the combined effect of other genotypes of *AGTR1* A1166C and *CYP2C9*∗3 was examined, but no difference was observed in the unadjusted or adjusted statistical model.

## 4. Discussion

Hypertension is one of the most common modifiable cardiovascular risk factors. The effective control of hypertension to reduce the morbidity and mortality of cardiovascular diseases is a global goal [25]. Interindividual variation in the BP response to the commonly used antihypertensive drug ARB is attributed to environmental and genetic factors, such as the polymorphisms in the genes involving targeted receptor and drug metabolism [14, 26, 27]. In the present study, we analyzed the association of polymorphisms *AGTR1* A1166C and *CYP2C9*∗3 with the antihypertensive effect of valsartan based on real-world data. High frequencies of the C allele and AC + CC genotype of *AGTR1* A1166C were observed in the well-controlled hypertensive patients and the AC + CC genotype was linked to a higher rate of hypertension control with valsartan treatment after adjustment for demographic and environmental factors (*P* = 0.018). These findings suggested that carriers with AC and CC genotypes exhibit an adequate antihypertensive efficacy response to valsartan, and the C allele of *AGTR1* A1166C may be an independent factor associated with good control of hypertension with valsartan treatment. However, the current study detected no significant difference in the allele or genotype distribution of *CYP2C9*∗3 polymorphism between well-controlled and poorly controlled patients. Moreover, *CYP2C9*∗3 polymorphism did not play a synergistic role in the effect of *AGTR1* A1166C polymorphism on the antihypertensive response to valsartan.

The polymorphism *AGTR1* A1166C located in the 3′ untranslated region of *AGTR1* may influence the stability of the mRNA expression and might be involved in the cellular signaling mediated by the angiotensin II receptor [28, 29]. Previous studies have shown that *AGTR1* A1166C is implicated in the hypertension risk and the sensitivity of ARBs; however, the conclusion is unanimous [14, 29–32]. Consistent with our findings, Sun et al. found that *AGTR1* A1166C was associated with the antihypertensive response of candesartan in a Chinese population, and individuals with the AC genotype had a significant reduction of SBP after candesartan medication [23]. Dong et al. also observed an enhanced antihypertensive effect of irbesartan only in male patients with AC and CC genotypes of *AGTR1* A1166C [33]. In another study on a large Chinese cohort (*n* = 1049), the decrease in BP was positively correlated with irbesartan concentration in the carriers with the C allele of *AGTR1* A1166C, suggesting an impact of the interaction of *AGTR1* polymorphism and irbesartan concentration on the antihypertensive response to irbesartan [27]. Conversely, the *AGTR1* 1166 C allele was not linked to the irbesartan concentration-BP response correlation in a Swedish study (*n* = 42). On the other hand, Konoshita et al. suggested that the *AGTR1* A1166C polymorphism was not a predictor of the response to antihypertensive treatment with valsartan [32].

Similarly, the results from previous studies on the association between *CYP2C9* polymorphism and the antihypertensive function of ARBs are contradictory. The *CYP2C9*∗3 polymorphism may lead to decreased activity of the CYP2C9 enzyme, which is widely studied in the Asian population [16–18, 34, 35]. The prodrug losartan is oxidized by the liver enzyme CYP2C9 and converted into an active form. Several studies implicated that the BP-lowering effect of losartan may be weakened in carriers with the *CYP2C9*∗3 polymorphism due to decreased activation of losartan [16, 35], while other ARBs, such as irbesartan and valsartan, need to be metabolized into inactive forms by the CYP2C9 enzyme. A previous study demonstrated that the patients with *CYP2C9* ∗1/∗3 or ∗3/∗3 genotype showed a significant decrease in SBP and DBP compared to patients with ∗1/∗1 genotype [33], which is different from our findings that no impact of *CYP2C9*∗3 polymorphism on the antihypertensive effect of valsartan was observed. Hiltunen et al. [22] suggested that the reproducibility of studies on the correlation between antihypertensive response and gene polymorphism was low. The study did not identify the association of *AGTR1* or *CYP2C9* polymorphisms with the antihypertensive effect of ARBs by using GWAS technology. Among the top 20 SNPs associated with losartan response in the GENRES study [36], few SNPs overlapped in the other studies conducted by Hiltunen et al. [22].

Herein, we found that age may be an independent factor for the response to valsartan treatment in our study, and patients with well-controlled hypertension were slightly older than the poorly controlled group, which was rarely reported in other studies. The factors, such as dietary habits, medication compliance, and physical activity, may be the underlying reasons that need to be explored further. Hypertension may be affected by many factors, such as age, race, and gene-gene interactions, which provide a multigenetic and multifactorial antihypertensive response of ARBs [18]. In addition, different sample sizes may also be a reason for the conflicting results of previous studies.

Nevertheless, the present study had some limitations. First, the sample size is limited leading to a limited statistical power to detect association. However, this retrospective study included patients with essential hypertension treated with valsartan in a large tertiary hospital during a 3-year period. The exclusion criteria were reasonable, and allele frequencies were in accordance with the Hardy–Weinberg equilibrium. Therefore, the study population was representative and the results were reliable. Second, factors such as dietary habits, patient compliance, and pharmacokinetics of ARBs among patients with different genotypes were not evaluated. Finally, this study only selected two common gene polymorphisms. Whether other gene polymorphisms are involved in the individual variability in the antihypertensive response to ARBs needs further investigation.

## 5. Conclusions

The current study showed that the polymorphism *AGTR1* A1166 C was significantly related to the antihypertensive effect of valsartan, suggesting that the C allele of *AGTR1* is an independent factor associated with better control of hypertension under valsartan treatment. The *CYP2C9*∗3 polymorphism was not significantly related to the antihypertensive response to valsartan in this study. Thus, further prospective studies with a large sample size are needed to clarify the association between gene polymorphisms and the antihypertensive efficacy response of ARBs.

## Figures and Tables

**Table 1:** Characteristics of hypertensive patients.

Variables	Well-controlled (*n* = 144)	Poorly controlled (*n* = 137)	*P*
Age (years)	55 (48, 62)	54 (44, 60)	0.012
Gender (male, %)	99, 68.8	104, 75.9	0.180
BMI (kg/m^2^)	26.5 (24.5, 29.6)	27.2 (25.2, 29.4)	0.276
History of smoking (*n*, %)	51, 35.4	63, 46.0	0.071
History of drinking (*n*, %)	53, 36.8	67, 48.9	0.040
Grade of hypertension (*n*, %)			0.001
Grade 1	22, 15.3	14, 10.2	
Grade 2	53, 36.8	28, 20.4	
Grade 3	69, 47.9	95, 69.3	
ALT (U/L)	24.0 (17.5, 34.5)	28.0 (19.0, 43.0)	0.032
TC (mmol/L)	4.3 (3.5, 5.0)	4.4 (3.6, 5.2)	0.286
TG (mmol/L)	1.6 (1.1, 2.2)	1.6 (1.2, 2.4)	0.279
LDL-C (mmol/L)	2.7 (2.1, 3.4)	2.9 (2.2, 3.6)	0.250
HDL-C (mmol/L)	1.0 (0.9, 1.2)	1.0 (0.8, 1.1)	0.045
Crea (*μ*mol/L)	69.9 (59.1, 82.0)	70.6 (62.7, 83.4)	0.383
eGFR (ml/min per 1.73 m^2^)	98.5 (89.4, 104.9)	101.5 (92.0, 107.0)	0.103

ALT: alanine aminotransferase; BMI: body mass index; Crea: creatinine; eGFR: estimated glomerular filtration rate; HDL-C: high-density lipoprotein cholesterol; LDL-C: low-density lipoprotein cholesterol; TC: total cholesterol; TG: triglyceride. Data are shown as median (interquartile range) or %. *P* < 0.05 indicates statistical significance.

**Table 2:** Genotype distribution and allele frequency of *AGTR1* A1166C and *CYP2C9*∗3 in well-controlled group (*n* = 144) and poorly controlled group (*n* = 137).

	Genotypes, *n* (%)				Alleles, *n* (%)		
*AGTR1* A1166C	AA	AC	CC	*P* ^a^	A	C	*P*
Well-controlled	119 (82.6)	24 (16.7)	1 (0.7)	0.006	262 (91.0)	26 (9.0)	0.005
Poorly controlled	128 (93.4)	9 (6.6	0 (0)		265 (96.7)	9 (3.3)	
*CYP2C9*∗3	∗1/∗1	∗1/∗3	∗3/∗3	*P* ^b^	∗1	∗3	*P*
Well-controlled	134 (93.1)	10 (6.9)	0 (0)	0.239	278 (96.5)	10 (3.5)	0.250
Poorly controlled	122 (89.1)	15 (10.9)	0 (0)		259 (94.5)	15 (5.5)	

^a^ *P* value was calculated for comparison between genotype AA and AC + CC using Chi-square test. ^b^*P* value was calculated for comparison between genotype ∗1/∗1 and ∗1/∗3 using chi-square test.

**Table 3:** Associations of gene polymorphisms and good response to valsartan treatment in the univariate and multivariate analyses.

	Unadjusted		Adjusted	
	OR (95% CI)	*P*	OR (95% CI)	*P*
*AGTR1*				
AA	1^a^	—	1^a^	—
AC + CC	2.988 (1.340–6.661)	0.007	2.764 (1.171–6.528)	0.020
*CYP2C9*				
∗1/∗1	1^a^	—	1^a^	—
∗1/∗3	0.607 (0.263–1.402)	0.242	0.565 (0.222–1.438)	0.231
Age	—	—	1.035 (1.004–1.067)	0.028
Gender	—	—	0.776 (0.379–1.589)	0.489
BMI	—	—	1.000 (0.924–1.083)	0.994
Grade of hypertension				
Grade 1	—	—	1^a^	—
Grade 2	—	—	1.005 (0.408–2.475)	0.991
Grade 3	—	—	0.513 (0.226–1.162)	0.110
History of drinking	—	—	0.650 (0.338–1.252)	0.198
History of smoking	—	—	0.752 (0.391–1.448)	0.394

^a^Reference category (odds ratio, 1). BMI: body mass index; CI: confidence interval; OR: odds ratio.

**Table 4:** Combined effect of *AGTR1* A1166C and *CYP2C9*∗3 polymorphisms on antihypertensive efficacy of valsartan.

	Unadjusted		Adjusted	
	OR (95% CI)	*P*	OR (95% CI)	*P*
Genotype (*AGTR1* + *CYP2C9*)				
AA + ∗1/∗1	1^a^	—	1^a^	—
AA + ∗1/∗3	0.587 (0.237–1.454)	0.250	0.626 (0.239–1.641)	0.341
(AC + CC) + ∗1/∗1	2.953 (1.267–6.880)	0.012	3.028 (1.225–7.486)	0.016
(AC + CC) + ∗1/∗1	2.054 (0.184–22.976)	0.559	0.606 (0.034–10.859)	0.734
Age	—	—	1.035 (1.004–1.067)	0.028
Gender	—	—	0.763 (0.372–1.566)	0.460
BMI	—	—	1.001 (0.925–1.085)	0.971
Grade of hypertension				
Grade 1	—	—	1^a^	—
Grade 2	—	—	1.050 (0.423–2.607)	0.917
Grade 3	—	—	0.516 (0.227–1.169)	0.113
History of drinking	—	—	0.629 (0.325–1.220)	0.170
History of smoking	—	—	0.770 (0.398–1.488)	0.436

^a^Reference category (odds ratio, 1). BMI: body mass index; CI: confidence interval; OR: odds ratio.

## Data Availability

Data used to support the findings of this study will be available upon reasonable request.

## References

[1] Lawes C. M., Vander Hoorn S., Rodgers A. (2008). International Society of Hypertension. Global burden of blood-pressure-related disease, 2001. *Lancet*.

[2] World Health Organization (2013). *A Global Brief on Hypertension: Silent Killer, Global Public Health Crisis: World Health Day 2013*.

[3] GBD 2017 Risk Factor Collaborators (2018). Global, regional, and national comparative risk assessment of 84 behavioural, environmental and occupational, and metabolic risks or clusters of risks for 195 countries and territories, 1990-2017: a systematic analysis for the Global Burden of Disease Study 2017. *Lancet*.

[4] Joint Committee for Guideline Revision (2019). 2018 Chinese guidelines for prevention and treatment of hypertension-a report of the revision committee of Chinese guidelines for prevention and treatment of hypertension. *Journal of Geriatric Cardiology*.

[5] Wang Z., Chen Z., Zhang L. (2018). Status of hypertension in China: results from the China hypertension survey, 2012-2015. *Circulation*.

[6] Mancia G., Messerli F., Bakris G., Zhou Q., Champion A., Pepine C. J. (2007). Blood pressure control and improved cardiovascular outcomes in the international verapamil SR-trandolapril study. *Hypertension*.

[7] Lee J. H., Kim S. H., Kang S. H. (2018). Blood pressure control and cardiovascular outcomes: real-world implications of the 2017 ACC/AHA hypertension guideline. *Scientific Reports*.

[8] Han T. S., Wang H. H., Wei L. (2017). Impacts of undetected and inadequately treated hypertension on incident stroke in China. *BMJ Open*.

[9] Zhang F.-L., Guo Z.-N., Xing Y.-Q., Wu Y.-H., Liu H.-Y., Yang Y. (2017). Hypertension prevalence, awareness, treatment, and control in northeast China: a population-based cross-sectional survey. *Journal of Human Hypertension*.

[10] Caldeira D., David C., Sampaio C. (2012). Tolerability of angiotensin-receptor blockers in patients with intolerance to angiotensin-converting enzyme inhibitors: a systematic review and meta-analysis. *American Journal of Cardiovascular Drugs*.

[11] Ross S. D., Akhras K. S., Zhang S., Rozinsky M., Nalysnyk L. (2001). Discontinuation of antihypertensive drugs due to adverse events: a systematic review and meta-analysis. *Pharmacotherapy: The Journal of Human Pharmacology and Drug Therapy*.

[12] Wu J., Du X., Lv Q. (2020). A phase 3 double-blind randomized (CONSORT-compliant) study of azilsartan medoxomil compared to valsartan in Chinese patients with essential hypertension. *Medicine*.

[13] Cui B., Dong Z., Zhao M. (2020). Analysis of adherence to antihypertensive drugs in Chinese patients with hypertension: a retrospective analysis using the China health insurance association database. *Patient Preference and Adherence*.

[14] Kurland L., Hallberg P., Melhus H. (2008). The relationship between the plasma concentration of irbesartan and the antihypertensive response is disclosed by an angiotensin II type 1 receptor polymorphism: results from the Swedish irbesartan left ventricular hypertrophy investigation vs. atenolol (SILVHIA) Trial. *American Journal of Hypertension*.

[15] Nantel P., René de Cotret P. (2010). The evolution of angiotensin blockade in the management of cardiovascular disease. *Canadian Journal of Cardiology*.

[16] Babaoglu M. O., Yasar U., Sandberg M. (2004). CYP2C9 genetic variants and losartan oxidation in a Turkish population. *European Journal of Clinical Pharmacology*.

[17] Hong X., Zhang S., Mao G. (2005). CYP2C9∗3 allelic variant is associated with metabolism of irbesartan in Chinese population. *European Journal of Clinical Pharmacology*.

[18] Chen K., Xiao P., Li G., Wang C., Yang C. (2021). Distributive characteristics of the CYP2C9 and AGTR1 genetic polymorphisms in Han Chinese hypertensive patients: a retrospective study. *BMC Cardiovascular Disorders*.

[19] Chen G., Jiang S., Mao G. (2006). CYP2C9 Ile359Leu polymorphism, plasma irbesartan concentration and acute blood pressure reductions in response to irbesartan treatment in Chinese hypertensive patients. *Methods and Findings in Experimental and Clinical Pharmacology*.

[20] Turner S. T., Schwartz G. L., Chapman A. B., Hall W. D., Boerwinkle E. (2001). Antihypertensive pharmacogenetics: getting the right drug into the right patient. *Journal of Hypertension*.

[21] Rysz J., Franczyk B., Rysz-Górzyńska M., Gluba-Brzózka A. (2020). Pharmacogenomics of hypertension treatment. *International Journal of Molecular Sciences*.

[22] Hiltunen T. P., Donner K. M., Sarin A. P. (2015). Pharmacogenomics of hypertension: a genome-wide, placebo-controlled cross-over study, using four classes of antihypertensive drugs. *Journal of American Heart Association*.

[23] Sun Y., Liao Y., Yuan Y. (2014). Influence of autoantibodies against AT1 receptor and AGTR1 polymorphisms on candesartan-based antihypertensive regimen: results from the study of optimal treatment in hypertensive patients with anti-AT1-receptor autoantibodies trial. *Journal of the American Society of Hypertension*.

[24] Williams B., Mancia G., Spiering W. (2018). 2018 ESC/ESH guidelines for the management of arterial hypertension: the task force for the management of arterial hypertension of the European society of cardiology and the European society of hypertension: the task force for the management of arterial hypertension of the European society of cardiology and the European society of hypertension. *Journal of Hypertension*.

[25] Mulerova T., Uchasova E., Ogarkov M., Barbarash O. (2020). Genetic forms and pathophysiology of essential arterial hypertension in minor indigenous peoples of Russia. *BMC Cardiovascular Disorders*.

[26] Johnson A. D., Newton-Cheh C., Chasman D. I. (2011). Association of hypertension drug target genes with blood pressure and hypertension in 86 588 individuals. *Hypertension*.

[27] Jiang S., Hsu Y.-H., Venners S. A. (2011). Interactive effect of angiotensin II type 1 receptor (AGT1R) polymorphisms and plasma irbesartan concentration on antihypertensive therapeutic responses to irbesartan. *Journal of Hypertension*.

[28] Braliou G. G., Grigoriadou A.-M. G., Kontou P. I., Bagos P. G. (2014). The role of genetic polymorphisms of the renin-angiotensin system in renal diseases: a meta-analysis. *Computational and Structural Biotechnology Journal*.

[29] Thekkumkara T. J., Linas S. L. (2003). Evidence for involvement of 3′-untranslated region in determining angiotensin II receptor coupling specificity to G-protein. *Biochemical Journal*.

[30] Kobashi G., Hata A., Ohta K. (2004). A1166C variant of angiotensin II type 1 receptor gene is associated with severe hypertension in pregnancy independently of T235 variant of angiotensinogen gene. *Journal of Human Genetics*.

[31] Wang J.-L., Li Xue L., Hao P.-P., Feng Xu F., Chen Y.-G., Yun Zhang Y. (2010). Angiotensin II type 1 receptor gene A1166C polymorphism and essential hypertension in Chinese: a meta-analysis. *Journal of the Renin-Angiotensin-Aldosterone System*.

[32] Konoshita T., Kato N., Fuchs S. (2009). Genetic variant of the renin-angiotensin system and diabetes influences blood pressure response to angiotensin receptor blockers. *Diabetes Care*.

[33] Dong H., Wang F.-z., Shi K., Zhang X.-s., Lv D.-m. (2021). Association of cytochrome P450 2C9∗3 and angiotensin II receptor 1 (1166A>C) gene polymorphisms with the antihypertensive effect of irbesartan. *American Journal of Hypertension*.

[34] Yu B. N., Luo C. H., Wang D. (2004). CYP2C9 allele variants in Chinese hypertension patients and healthy controls. *Clinica Chimica Acta; International Journal of Clinical Chemistry*.

[35] Sekino K., Kubota T., Okada Y. (2003). Effect of the single CYP2C9∗3 allele on pharmacokinetics and pharmacodynamics of losartan in healthy Japanese subjects. *European Journal of Clinical Pharmacology*.

[36] Hiltunen T., Suonsyrja T., Hannilahandelberg T. (2007). Predictors of antihypertensive drug responses: initial data from a placebo-controlled, randomized, cross-over study with four antihypertensive drugs (The GENRES Study). *American Journal of Hypertension*.

